# Demographic, Husbandry, and Biosecurity Factors Associated with the Presence of *Campylobacter* spp. in Small Poultry Flocks in Ontario, Canada

**DOI:** 10.3390/pathogens10111471

**Published:** 2021-11-12

**Authors:** Paige M. Schweitzer, Leonardo Susta, Csaba Varga, Marina L. Brash, Michele T. Guerin

**Affiliations:** 1 Department of Population Medicine, Ontario Veterinary College, University of Guelph, Guelph, ON N1G 2W1, Canada; mguerin@uoguelph.ca; 2 Department of Pathobiology, Ontario Veterinary College, University of Guelph, Guelph, ON N1G 2W1, Canada; lsusta@uoguelph.ca; 3 Ontario Ministry of Agriculture, Food and Rural Affairs, Guelph, ON N1G 2W1, Canada; cvarga@illinois.edu; 4 Animal Health Laboratory, University of Guelph, Guelph, ON N1G 2W1, Canada; mbrash@uoguelph.ca

**Keywords:** backyard flock, management, poultry housing, *Campylobacter* species, epidemiology, cross-sectional study, biosecurity practices, chicken, turkey

## Abstract

This study is part of a 2 year disease surveillance project conducted to establish the prevalence of poultry and zoonotic pathogens, including *Campylobacter* spp., among small poultry flocks in Ontario, Canada. For each post-mortem submission to the Animal Health Laboratory, a pooled sample of cecal tissue was cultured for *Campylobacter* spp., and a husbandry and biosecurity questionnaire was completed by the flock owner (*n* = 153). Using both laboratory and questionnaire data, our objective was to investigate demographic, husbandry, and biosecurity factors associated with the presence of *Campylobacter* spp. in small flocks. Two multivariable logistic regression models were built. In the farm model, the odds of *Campylobacter* spp. were higher in turkeys, and when birds were housed in a mixed group with different species and/or types of birds. The odds were lower when antibiotics were used within the last 12 months, and when birds had at least some free-range access. The effect of pest control depended on the number of birds at risk. In the coop model, the odds of *Campylobacter* spp. were lower when owners wore dedicated clothing when entering the coop. These results can be used to limit the transmission of *Campylobacter* spp. from small poultry flocks to humans.

## 1. Introduction

Globally, raising small poultry flocks (hereafter referred to as *small flocks*) in urban, semi-urban, and rural communities has increased in popularity [1,2]. This trend has also been observed in Canada, with over 16,000 small flocks registered in the province of Ontario alone in 2016 [3]. In Canada, the commercial production of chicken and turkey products, table eggs, and broiler hatching eggs is controlled by the supply management system [4], and each province sets its own limits for non-commercial production. In Ontario, residents may keep up to 99 laying hens, 300 broiler chickens, and 50 turkeys per premise without quota [4]. Ducks, pheasants, quail, and other domestic poultry species are not regulated by supply management; therefore, there is no quota limit for these species [3,5].

Despite the increase in popularity, little is known about the effect of husbandry and biosecurity practices on the presence of poultry and zoonotic pathogens in small flocks. Although studies have been conducted on small flock demographics, husbandry practices of flock owners, and the prevalence and epidemiologic characterization of avian influenza and *S. enterica* [1,6,7,8,9,10,11,12,13,14], none have investigated risk factors for the presence of *Campylobacter* spp. Husbandry and biosecurity are often inadequate in small flocks [7,9,13,15], leading to concerns regarding the risk of transmission of zoonotic pathogens, such as *Campylobacter* spp., from small flocks to humans. Furthermore, birds in small flocks are often treated as pets [7,16], leading to close contact (e.g., increased handling, petting) that could increase the risk of exposure to zoonotic pathogens. Such exposure can have significant consequences for individuals at higher risk of serious infections (e.g., pregnant women, children) [17].

*Campylobacter jejuni* and *Campylobacter coli* are causes of diarrhea in humans [18]. Improper handling of contaminated food and consumption of undercooked food, in particular poultry products, and direct contact with livestock and pets, are major risk factors for *C. jejuni* and *C. coli* infections in humans [19,20]. Indeed, poultry is a primary food-related source of *Campylobacter* spp. to humans, causing an estimated 50 to 70% of campylobacteriosis cases [21,22]. Contaminated feed, water, and fomites, as well as wild birds, rodents, and insects, are sources of *Campylobacter* spp. in poultry [23]. Once introduced into a flock, *Campylobacter* spreads rapidly, colonizing the intestinal tract of broiler chickens within 1 week, with the chickens remaining colonized until slaughter [24,25]. *Campylobacter* spp. shed by poultry through their feces can be spread throughout the environment, contaminating feed, tools, and other items that come into contact with humans [7,16,26].

To establish the prevalence of poultry and zoonotic pathogens and poultry diseases among small flocks in Ontario, a cross-sectional, disease surveillance project was conducted from October 2015 to September 2017. Birds submitted to the Animal Health Laboratory (AHL), University of Guelph for post-mortem examination were also tested for an array of pathogens using pre-set microbiology tests. Furthermore, for each submission, the flock owner was required to complete a husbandry and biosecurity questionnaire. Results of the surveillance project are presented in three companion papers [15,16,27]. *Campylobacter* spp. (*C. jejuni* and *C. coli*) were isolated from 35% of 158 tested submissions [16]. This is higher than the prevalence estimate from the 2012–2013 National Microbiological Baseline Study, which found that 20% of commercial broiler chicken lots sampled at federally registered slaughter plants in Ontario tested positive for *Campylobacter* spp. [28]. Thus, the objective of this study was to investigate demographic, husbandry, and biosecurity factors associated with the presence of *Campylobacter* spp. in small flocks in Ontario.

## 2. Results

### 2.1. Campylobacter

The species breakdown of *Campylobacter* has been described previously [16]. In brief, *C. jejuni* and *C. coli* were detected in 18% and 20% of tested submissions, respectively. Chickens accounted for 84% of submissions, with approximately equal proportions of *C. jejuni* and *C. coli*. The majority (86%) of the *Campylobacter*-positive turkey submissions were *C. coli*.

### 2.2. Description of Questionnaire Data

The questionnaire had 41 questions [15]; however, for many questions, flock owners could check more than one box or provide additional information (such that the responses were not mutually exclusive), resulting in more variables than questions after data management (see Materials and Methods). Some questions were not applicable to free-range flocks; thus, two separate models were built. The farm model utilized data from 153 flocks with all housing types and investigated 44 demographic, husbandry, and biosecurity variables; these originated from 24 questions. The coop model included 124 flocks with the housing types being either inside only or inside with some free-range access, and investigated 15 indoor housing-related variables; these originated from eight questions. Data from the remaining questions lacked variability, were not considered to be reliable, or were not considered to be relevant for *Campylobacter*.

### 2.3. Farm Model

There were 44 demographic, husbandry, and biosecurity variables analyzed in the univariable analysis for the farm model (Table 1 and Table 2). Variables that met the screening criterion (*p* ≤ 0.2) on univariable analysis are shown in bold, in Table 1 and Table 2. However, due to collinearity, not all of those were offered to the multivariable model; of the two nested and highly correlated variables pertaining to pest control, the non-specific pest control variable (one or more pest control measures were in place for rodents, flies, beetles and other pests) was selected for further analysis, as it had the lowest *p* value.

The final multivariable model included: species: turkey; the number of birds at risk; pest control; an interaction between the number of birds at risk and pest control; medication: antibiotics; bird housing type; and mixed group (Table 3). The odds of *Campylobacter* spp. presence were higher in turkeys compared to other species (OR = 16.89, *p* = 0.044) and when birds were housed in a mixed group with different species (e.g., turkeys and chickens) and/or types (e.g., broilers and layers) of birds (OR = 4.61, *p* = 0.004). The odds of *Campylobacter* spp. presence were lower when antibiotics were used within the last 12 months (OR = 0.19, *p* = 0.004) and when birds were housed inside with some free-range access (OR = 0.28, *p* = 0.017) compared to when birds were housed indoors only; a similar, albeit non-significant trend was identified when birds were exclusively free-range (OR = 0.29, *p* = 0.067). The effect of pest control on the presence of *Campylobacter* spp. depended on the number of birds at risk: the odds were lower in larger flocks (50–260 birds) in which pest control was used compared to smaller flocks (1–24 birds) in which pest control was not used (OR = 0.04, *p* = 0.030). All statistically significant *(p* ≤ 0.05) contrasts for the number of birds at risk and pest control interaction are presented in Table 4.

The Pearson chi-square goodness-of-fit test indicated that the model fit the data (Χ^2^ = 40.85, *p* = 0.608). In the final model, there were two observations (belonging to the same covariate pattern) with large, negative, standardized residuals (−3.7) that were considered to be outliers and had a relatively large influence on the model. These were medium-sized flocks (25–49 birds) of chickens in which pest control was used; they were housed indoors in a mixed group and were not given antibiotics within the last 12 months, yet were *Campylobacter*-negative. When removed from the model, there were no major changes to any of the coefficients, so they were kept in the final model.

### 2.4. Coop Model

There were 15 indoor housing-related variables analyzed in the univariable analysis for the coop model (Table 5). Variables that met the screening criterion (*p* ≤ 0.2) on univariable analysis are shown in bold, in Table 5. However, due to collinearity, not all of those were offered to the multivariable model; of the six nested and highly correlated variables pertaining to the use of dedicated shoes and/or clothing when entering and/or cleaning the coop, wearing dedicated clothing when entering the coop was selected for further analysis, as it had the lowest *p* value.

After the backward elimination process was completed, the final model (*n* = 111) included only one significant variable: wearing dedicated clothing when entering the coop (OR = 0.23, 95% CI = 0.07–0.73, *p* = 0.012).

## 3. Discussion

*Campylobacter* spp. were detected in more than one third of the tested submissions in our study population [16], highlighting the importance of understanding the epidemiology of this zoonotic pathogen in this sector of the poultry industry. We identified several demographic characteristics, husbandry practices, and biosecurity measures associated with the presence of *Campylobacter* spp. that can help small flock owners implement effective prevention and control measures to limit the zoonotic transmission of *Campylobacter* spp. from poultry to humans.

We found that the risk of *Campylobacter* spp. was higher in turkey submissions compared to submissions of other poultry species (predominantly chickens). Although commercial broiler chicken flocks are recognized as an important reservoir of *Campylobacter* spp., our finding suggests that turkeys can also be a reservoir and agrees with previous studies conducted on commercial turkey flocks. In Québec, Canada, the prevalence of *Campylobacter*-positive turkey flocks was 46% [29], and in Italy, all three flocks tested were positive for *Campylobacter* spp. [30]. Although there were relatively few turkey submissions in our study, 70% of them were positive for *Campylobacter* spp. (mainly *C. coli*) [16], stressing the need for small flock owners to take precautions when handling their turkeys or consuming their products.

We found that the risk of *Campylobacter* spp. was lower when antibiotics were used in the flock within the last 12 months, suggesting a potential relationship between antibiotic use and the gut microflora of the birds. The details of their use were not well-described by the flock owners, although tetracycline, tylosin, and penicillin were among those reported [15]. At the time of the study, flock owners could purchase antibiotics for their birds without a prescription at feed mills, co-ops, and farm supply stores. However, antimicrobial use regulations were updated in Canada effective 1 December 2018, such that a veterinary prescription is now required for all medically important antimicrobials in human medicine [31]. Although there is limited knowledge on the use of antibiotics in small flocks, it is well known that overuse and misuse of antibiotics in food animals is a contributing factor for the emergence of antimicrobial-resistant enteric bacteria, such as *Campylobacter* spp. [32]. A high proportion of the *C. jejuni* and *C. coli* isolates in our study population were resistant to tetracycline [32], warranting further work on investigating the effect of husbandry and biosecurity on antimicrobial resistance in the *C. jejuni* and *C. coli* isolates.

Our study found that housing factors likely play a role in the occurrence of *Campylobacter* spp. in small flocks. The risk of *Campylobacter* spp. was higher when birds were housed in a mixed group, and lower when birds had at least some free-range access compared to when housed indoors only. It is well known that poultry species, including chickens, turkeys, and ducks, are reservoirs of *Campylobacter* spp.; therefore, housing different poultry species together may lead to transmission between species through environmental contamination or direct contact [33,34]. In addition, restricting birds to an indoor space could result in an accumulation of viable bacteria in the coop, thereby increasing the risk of infection in these flocks. Our finding differs from a study conducted on commercial poultry in the United Kingdom, in which the prevalence of *Campylobacter* spp. was higher in the free-range flocks and broiler chickens with outdoor access compared to conventionally-raised flocks [35]. Poultry that have access to the outdoors have increased contact with the environment and with a large number of possible sources of infection. Farm animals, pets, and wild birds, such as ducks, turkeys, gulls, and pigeons, are known to be carriers of *Campylobacter* spp. [36,37,38,39]. Furthermore, soil in the area around commercial poultry houses is a potential source of *Campylobacter* spp. contamination [40]. This indicates that housing factors associated with *Campylobacter* spp. in small flocks may be very different from those in commercial flocks.

In our study, the effect of pest control on the presence of *Campylobacter* spp. depended on the number of birds at risk: the odds were lower in larger flocks that used pest control compared to smaller flocks that did not use pest control. Indeed, the results of our comparisons indicate that for larger flocks, having pest control methods in place is especially important to reduce the risk of *Campylobacter*. There is limited knowledge on the relationship between flock size and pest control in small flocks; however, pests commonly found on livestock premises, including rodents and insects, are known vectors of *Campylobacter* spp. [23,41]. Interestingly, our findings differ from a study conducted in Québec, in which the odds of colonization with *Campylobacter* spp. were found to be higher for commercial chicken flocks with professional rodent control compared to those without rodent control [29]. Larger flock size in commercial broiler flocks is associated with higher odds of *Campylobacter* spp. [38,42]; however, commercial flocks are substantially larger and managed differently from small flocks, making it difficult to compare studies.

In our coop model, we found that the risk of *Campylobacter* spp. was lower for flocks in which the owner reported wearing dedicated clothing when entering the coop. This agrees with studies conducted on commercial broiler flocks, which showed that dedicated personal protective equipment (clothing) reduced the prevalence of *Campylobacter* spp. on farm staff and transporters [43,44]. However, other studies on commercial broiler chicken farms found that the use of dedicated clothing did not have a significant influence on the *Campylobacter* status of the flock [38,45,46].

The questionnaire included several questions related to potential environmental sources of *Campylobacter* spp. that might be unique to small flocks, including the disposal method for dead birds, the presence of a wild bird feeder on the property, having a body of water on the property, and having additional domestic animals present on the property. However, only a few were significant on univariable analysis and none were significant on multivariable analysis. Overall, small flocks are very different from commercial flocks, making comparisons difficult. Small flocks have a different composition than commercial flocks, often with different bird species, breeds, and ages housed together. The average flock size in our study was 25 birds, whereas the average size of commercial layer and chicken flocks in Canada is approximately 22,000 and 14,000 birds, respectively [47,48]. Furthermore, small flocks can have different husbandry and biosecurity practices, as well as different environmental exposures, such as outdoor access [1,2,9,15]. Organic production makes up a relatively small proportion of commercial production in Canada, and outdoor access for these flocks is weather-permitting and usually limited to the spring, summer, and fall. Globally, *Campylobacter* spp. presence in commercial flocks has been associated with numerous flock, barn, farm, and environmental exposures. Flock-level factors include: flock age; flock size; stocking density; antibiotic treatment; and feeding program [26,38,42,45,46]. Barn-level factors include: barn age; barn design; ventilation system design; water line system design; footwear management; rodent control; biosecurity; cleaning and disinfection; downtime; and flock thinning [26,29,38,46,49]. Farm-level factors include: farm size and geographic location; water source and treatment; feed storage; manure storage; manure spreading; and the presence/density of other poultry and livestock on the farm and in the region [26,29,45,46,50,51]. Environmental factors include: flies; temperature; and rainfall [41,49,50]. Our study demonstrates that some factors associated with the presence of *Campylobacter* spp. in small flocks are similar to those for commercial flocks (e.g., flock size), whereas other factors (e.g., mixed groups) are indeed unique.

A potential limitation of this study is sampling bias, as a relatively higher number of submissions were received from areas close to the two AHL locations in Guelph and Kemptville [16]. However, we assume this bias to be small, because submissions were received from most areas of the province [16]. Non-differential misclassification of independent variables (i.e., misclassification unrelated to flock *Campylobacter* status) might have occurred due to errors in reporting by flock owners or grouping of categories, potentially resulting in conservative estimates of risk. Additionally, the submissions were all from sick or dead birds, and the occurrence of *Campylobacter* spp. might differ in healthy flocks. Future zoonotic disease surveillance studies that include healthy flocks would add valuable insight into our understanding of the epidemiology of this zoonotic pathogen in this sector of the poultry industry.

## 4. Materials and Methods

### 4.1. Study Design

This study was part of a larger, cross-sectional, small-flock disease surveillance project that took place between October 2015 and September 2017. The project details and inclusion criteria have been described previously [16]. In brief, Ontario small flock owners were encouraged to submit up to five of their sick or deceased birds to the AHL for a post-mortem evaluation and diagnostic investigation at a subsidized cost, to determine the cause(s) of morbidity or mortality of the submitted birds. In addition, pre-set microbiology tests were conducted on pooled samples from all of the birds in each submission to detect flock infection, regardless of clinical history or post-mortem findings. Isolation for *Campylobacter* spp. was conducted on pooled cecal tissues. Each pooled cecal sample (one per submission) consisted of cecal tissue from 1–5 birds (median 1, mean 1.3), originating from flocks ranging in size from 1–299 birds (median 25, mean 26) [16]. All tests were conducted in accordance with the AHL’s standard operating procedures. The AHL is an American Association of Veterinary Laboratory Diagnosticians-accredited diagnostic facility that serves as the provincial animal health laboratory for Ontario. Samples were directly plated on modified charcoal, cefoperazone, deoxycholate selective agar (Bio-Media Unlimited Ltd., Toronto, ON, Canada) and incubated in a microaerophilic environment at 37 °C for 72 h [52]. Colonies resembling *Campylobacter* spp. were identified using matrix-assisted laser desorption ionization time-of-flight mass spectrometry (MALDI-TOF) (Bruker Ltd., Billerica, MA, USA) using a direct transfer method. Briefly, bacterial colonies were streaked on the stainless-steel target plate and overlaid with 1 μL of alpha-cyano-4-hydroxycinnamic acid (HCCA) [53]. Flock owners had to complete a consent form and a paper-based husbandry and biosecurity questionnaire before or at the time of bird submission, in order to participate in the project.

### 4.2. Questionnaire

The details regarding the questionnaire design and data management have been described previously [15]. In brief, the questionnaire included 41 questions, 27 of which were binomial, 1 of which was open-ended, and 13 of which were multiple choice. Of the 13 multiple-choice questions, 12 were not mutually exclusive, in that participants could select multiple options. Furthermore, 24 of the multiple-choice and binomial questions were semi-closed, in that participants could add additional information as needed. The questionnaire was composed of two sections. The first section pertained to the birds being submitted, and it was comprised of questions regarding flock and housing characteristics, husbandry, biosecurity, vaccination, and medication use. The second section of the questionnaire focused on the general premises: the presence of other domestic animals; source, treatment, and bacterial testing of the drinking water; and whether any household members worked with commercial poultry.

For multiple-choice questions that were not mutually exclusive, all checked answers were tallied for each questionnaire; these were converted to individual, dichotomous variables in the analyses. Answers to questions pertaining to an indoor coop or barn were only tallied if the owner responded that the flock had some indoor access. The questionnaires had various numbers of unanswered and/or incomplete questions. As a result, the number of valid answers for each question varied.

### 4.3. Data Management

#### 4.3.1. Farm Model

A farm model was built to investigate demographic, husbandry, and biosecurity factors associated with the presence of *Campylobacter* spp. For this model, the number of birds at risk reported on the AHL post-mortem submission form was used, as these data were considered to be more complete (*n* = 140) and reliable than data from the questionnaire pertaining to the number of birds of each production type.

There were three questions on the questionnaire pertaining to drinking water for the flock: source (municipal, well, pond); whether the water was treated; and whether the water had been tested for bacteria. These were combined into a summary variable: drinking water risk level. Drinking water risk level was categorized as low risk (municipal water), medium–low risk (well water that had been treated and/or tested for bacteria), medium risk (well water that had been neither treated nor tested for bacteria), and high risk (pond water). The isolation of new birds risk level variable was a summary variable derived from the isolation of new birds (yes/no) and the isolation duration variables to simplify the various responses given to the questions. It was categorized as low risk (isolating for >2 weeks or all-in-all-out), medium risk (isolating for ≤2 weeks or isolating for an unknown amount of time), and high risk (no isolation).

The continuous variables, number of birds at risk and the period of time the flock was present on the owner’s property, did not meet the linearity assumption and a quadratic term was not appropriate, so the variables were categorized using Lowess curves (described below). The number of birds at risk was categorized as 1–24 birds (smaller flocks), 25–49 birds (medium-sized flocks), and 50–260 birds (larger flocks). The period of time the flock was present on the owner’s property was categorized as 0–19 months, 20–59 months, and ≥60 months.

#### 4.3.2. Coop Model

A coop model was built to investigate indoor housing-related factors associated with the presence of *Campylobacter* spp. The barn cleaning and/or disinfection variable had three categories: fairly frequently (when flock owners reported cleaning after each flock or cleaning more than once a year); infrequently (when flock owners reported cleaning once a year, less than once a year, or as needed); and not applicable (when flock owners reported that it was a new coop, their first flock and/or birds, or not done yet). The category ‘not applicable’ was excluded from the analysis because we were only interested in barns that had been cleaned and/or disinfected. Likewise, the ‘as needed’ category of the frequency of removing soiled litter and/or fecal material variable was excluded because it could have been interpreted in different ways by different flock owners, and the response of ‘never’ was combined with ‘yearly’ to eliminate a category that had only one response. There were several questions on the questionnaire pertaining to the use of dedicated shoes and clothing. These data were analyzed in a number of ways. First, as separate variables: wearing dedicated shoes when entering the coop; wearing dedicated shoes when cleaning the coop; wearing dedicated clothing when entering the coop; and wearing dedicated clothing when cleaning the coop. Next, by combining the shoes and clothing variables: wearing any personal protective equipment (**PPE**) when entering the coop; and wearing any PPE when cleaning the coop. Lastly, as a single, combined variable: wearing any PPE when entering and/or cleaning the coop. Flock owners were asked two questions pertaining to visitors: did they allow visitors into the coop (yes/no); and, if yes, were guests required to wear dedicated clothing. These data were analyzed in two different ways. First, as a simple, dichotomous variable: visitors were allowed (yes/no). Second, by combining responses from both questions into a summary variable with three categories: visitors were allowed into the coop and were required to wear dedicated clothing; visitors were allowed into the coop and were not required to wear dedicated clothing; and visitors were not allowed.

### 4.4. Statistical Analysis

Laboratory and questionnaire data were entered manually into Microsoft Office Excel 2016 (Microsoft Corporation, Redmond, WA, USA), where they were visually inspected for errors and coded. The data were then imported into STATA IC 16 (StataCorp, College Station, TX, USA) for statistical analyses.

Univariable logistic regression models were created to screen independent variables. Variables with insufficient variability (less than 10%) were excluded from the analysis, while variables that had a *p*-value ≤ 0.20 on univariable analysis were considered for further analysis. Lowess curves were used to assess linearity between the log odds of the outcome (presence/absence of *Campylobacter* spp.) and continuous variables that met the screening criterion. If the linearity assumption was not met, and a quadratic term was not appropriate, the variable was categorized based on cut points observed on the Lowess curve and then screened again. All pairwise correlations between independent variables that met the screening criterion were examined. When two variables were deemed to be highly correlated (rho ≥ |0.8|), *p* values, Akaike information criterion values, and biological plausibility were used to decide which variable would be offered to a multivariable model.

Significant, non-correlated variables from the univariable analysis were offered to a multivariable logistic regression model. To build the model, a manual backward selection method was used, with a *p*-value of ≤0.05 (Wald’s test for dichotomous and continuous variables, likelihood ratio test for categorical variables) indicating significance. When removed, if a variable changed the coefficient of any significant variable by ≥20%, it was considered to be a confounding variable and kept in the model regardless of statistical significance if the relationship was thought to be biologically plausible. Once a main effects model had been established, all possible two-way interactions were generated and assessed using the likelihood ratio test and Akaike information criterion. Using the *lincom* command in STATA, contrasts were built between interacting variables for all significant interaction terms.

To assess the fit of the model, a Pearson chi-square goodness-of-fit was conducted, and if the *p*-value was >0.05, we accepted that the model fit the data. Standardized Pearson residuals that were ≥|3.0| SDs were considered to be outliers, and the raw data were checked for errors, corrected if necessary, and the model refit. If there were no errors in the raw data, the outliers were kept in the model. Influential observations were investigated, removed from the model, and the model was refit to determine if their removal resulted in any significant changes. However, regardless of their impact, the influential observations were kept in the final model.

## 5. Conclusions

Poultry are reservoirs of *Campylobacter* spp., and although the birds are generally asymptomatic, it is an important zoonotic pathogen. We identified turkeys as having a higher risk of *Campylobacter* spp. than other poultry species, and described other demographic, husbandry, and biosecurity factors that were significantly associated with the presence of this pathogen in small flocks in Ontario. Our findings underline the importance of appropriate food safety and disease management methods by small flock keepers to prevent and control the zoonotic transmission of *Campylobacter* spp. via contact with infected poultry or ingestion of contaminated poultry meat.

## Figures and Tables

**Table 1:** Farm model: Univariable analysis of demographic variables in a study investigating factors associated with the presence of *Campylobacter* spp. in small poultry flocks in Ontario, Canada. Variables with a *p*-value of ≤0.2 (shown in bold) were considered for further analyses.

Variable Description	Category	OR	95% CI	*p*-Value
Species: chicken (*n* = 153)	No (*n* = 23)	Referent		
	Yes (*n* = 130)	0.62	0.25–1.53	0.3
Species: turkey (*n* = 153)	No (*n* = 144)	Referent		
	Yes (*n* = 9)	4.26	1.02–17.80	**0.047**
Species: waterfowl (*n* = 153)	No (*n* = 145)	Referent		
	Yes (*n* = 8)	0.63	0.12–3.25	0.584
Species: game bird (*n* = 153)	No (*n* = 145)	Referent		
	Yes (*n* = 8)	1.18	0.27–5.12	0.83
Production type: broiler (*n* = 151)	No (*n* = 134)	Referent		
	Yes (*n* = 17)	2.38	0.86–6.60	**0.095**
Production type: breeder (*n* = 151)	No (*n* = 138)	Referent		
	Yes (*n* = 13)	0.54	0.14–2.07	0.373
Production type: layer (*n* = 150)	No (*n* = 52)	Referent		
	Yes (*n* = 98)	0.6	0.30–1.21	**0.154**
Production type: dual-purpose (*n* = 151)	No (*n* = 133)	Referent		
	Yes (*n* = 18)	1.62	0.60–4.39	0.344
Number of birds at risk ^1^ (*n* = 140)	1–24 birds (*n* = 78)	Referent		
	25–49 birds (*n* = 32)	1.86	0.78–4.41	**0.161**
	50–260 birds (*n* = 30)	2.71	1.13–6.50	**0.025**
Reason for raising: personal consumption of meat or eggs (*n* = 153)	No (*n* = 47)	Referent		
	Yes (*n* = 106)	0.76	0.37–1.56	0.454
Reason for raising: farm gate sales (*n* = 153)	No (*n* = 126)	Referent		
	Yes (*n* = 27)	2.49	1.07–5.81	**0.034**
Reason for raising: breeding stock (*n* = 153)	No (*n* = 136)	Referent		
	Yes (*n* = 17)	0.56	0.17–1.83	0.339
Reason for raising: pet (*n* = 153)	No (*n* = 95)	Referent		
	Yes (*n* = 58)	1.04	0.52–2.06	0.919
Source of birds: hatchery (*n* = 152)	No (*n* = 93)	Referent		
	Yes (*n* = 59)	0.67	0.33–1.35	0.265
Source of birds: feed store (*n* = 152)	No (*n* = 128)	Referent		
	Yes (*n* = 24)	0.95	0.38–2.40	0.921
Source of birds: friends/neighbours (*n* = 152)	No (*n* = 105)	Referent		
	Yes (*n* = 47)	0.65	0.31–1.37	0.256
Period flock was present on owner’s property ^1^ (*n* = 151)	0–19 months (*n* = 109)	Referent		
	20–59 months (*n* = 31)	0.27	0.10–0.77	**0.014**
	60+ months (*n* = 11)	0.14	0.02–1.15	**0.067**

Abbreviations: OR, odds ratio; CI, confidence interval. ^1^ Indicates a continuous variable that has been categorized.

**Table 2:** Farm model: Univariable analysis of husbandry and biosecurity variables in a study investigating factors associated with the presence of *Campylobacter* spp. in small poultry flocks in Ontario, Canada. Variables with a *p*-value of ≤0.2 (shown in bold) were considered for further analyses.

Variable Description	Category	OR	95% CI	*p*-Value
Mixed housing with other animals (*n* = 145)	No (*n* = 90)	Referent		
	Yes (*n* = 55)	1.4	0.70–2.82	0.342
Mixed group with different species and/or types of birds (*n* = 153)	No (*n* = 102)	Referent		
	Yes (*n* = 51)	1.82	0.91–3.66	**0.093**
Bird housing (*n* = 153)	Inside only (*n* = 46)	Referent		
	Inside with some free-range access (*n* = 79)	0.24	0.11–0.53	**<0.001**
	Free-range only (*n* = 28)	0.26	0.09–0.72	**0.01**
Pest control: any method ^1^ (*n* = 153)	No (*n* = 70)	Referent		
	Yes (*n* = 83)	0.43	0.22–0.84	**0.014**
Pest control: rodent control (*n* = 153)	No (*n* = 118)	Referent		
	Yes (*n* = 35)	1.02	0.46–2.25	0.966
Pest control: insect control (*n* = 153)	No (*n* = 128)	Referent		
	Yes (*n* = 25)	0.56	0.21–1.51	0.254
Pest control: physical barrier (*n* = 153)	No (*n* = 133)	Referent		
	Yes (*n* = 20)	0.44	0.14–1.40	**0.165**
Feed kitchen waste or leftovers (*n* = 150)	No (*n* = 52)	Referent		
	Yes (*n* = 98)	0.34	0.17–0.70	**0.003**
Disposal method for dead birds: incineration (*n* = 152)	No (*n* = 119)	Referent		
	Yes (*n* = 33)	0.95	0.42–2.15	0.904
Disposal method for dead birds: burial (*n* = 152)	No (*n* = 93)	Referent		
	Yes (*n* = 59)	0.67	0.33–1.35	0.265
Disposal method for dead birds: manure pile (*n* = 152)	No (*n* = 132)	Referent		
	Yes (*n* = 20)	2.71	1.04–7.05	**0.041**
Disposal method for dead birds: composting (*n* = 152)	No (*n* = 128)	Referent		
	Yes (*n* = 24)	1.8	0.74–4.35	**0.195**
Handwashing before contact with flock (*n* = 143)	No (*n* = 74)	Referent		
	Yes (*n* = 69)	1.18	0.59–2.38	0.638
Handwashing after contact with flock (*n* = 146)	No (*n* = 9)	Referent		
	Yes (*n* = 137)	0.57	0.15–2.24	0.422
Isolation of new birds risk level ^2^ (*n* = 144)	Low risk (*n* = 51)	Referent		
	Medium risk (*n* = 57)	0.66	0.30–1.46	0.302
	High risk (*n* = 36)	0.99	0.41–2.37	0.975
Isolation of sick birds (*n* = 147)	No (*n* = 31)	Referent		
	Yes (*n* = 116)	0.67	0.30–1.52	0.342
Medication use within the last 12 months: antibiotics (*n* = 151)	No (*n* = 95)	Referent		
	Yes (*n* = 56)	0.5	0.24–1.04	**0.063**
Presence of a wild bird feeder on the property (*n* = 151)	No (*n* = 73)	Referent		
	Yes (*n* = 78)	1.08	0.55–2.12	0.821
Poultry feed and/or water accessible to rodents/wild animals/wild birds (*n* = 152)	No (*n* = 94)	Referent		
	Yes (*n* = 58)	0.73	0.36–1.48	0.385
Body of water on property accessible to poultry (*n* = 152)	No (*n* = 123)	Referent		
	Yes (*n* = 29)	1.05	0.45–2.47	0.906
Drinking water risk level ^3^ (*n* = 147)	Low risk (*n* = 25)	Referent		
	Medium-low risk (*n* = 90)	1.81	0.62–5.30	0.282
	Medium risk (*n* = 27)	3.71	1.08–12.80	**0.038**
	High risk (*n* = 5)	2.67	0.35–20.51	0.346
Cattle on property (*n* = 153)	No (*n* = 129)	Referent		
	Yes (*n* = 24)	2.23	0.92–5.38	**0.076**
Horses on property (*n* = 153)	No (*n* = 104)	Referent		
	Yes (*n* = 49)	1.77	0.87–3.57	**0.114**
Sheep and/or goats on property (*n* = 153)	No (*n* = 123)	Referent		
	Yes (*n* = 30)	1.65	0.72–3.72	0.231
Pigs on property (*n* = 153)	No (*n* = 125)	Referent		
	Yes (*n* = 28)	1.91	0.83–4.40	**0.128**
Domestic cats on property (*n* = 153)	No (*n* = 54)	Referent		
	Yes (*n* = 99)	0.49	0.25–0.99	**0.045**
Dogs on property (*n* = 153)	No (*n* = 37)	Referent		
	Yes (*n* = 116)	1.4	0.70–2.82	0.342

Abbreviations: OR, odds ratio; CI, confidence interval. ^1^ Pest control: one or more pest control measures were in place for rodents, flies, beetles, and other pests. ^2^ Isolation of new birds risk level was categorized as low risk (isolating for >2 weeks or all-in-all-out), medium risk (isolating for ≤2 weeks or isolating for an unknown amount of time), and high risk (no isolation). ^3^ Drinking water risk level was categorized as low risk (municipal water), medium–low risk (well water that had been treated and/or tested for bacteria), medium risk (well water that had been neither treated nor tested for bacteria), and high risk (pond water).

**Table 3:** Farm model: Multivariable logistic regression model of demographic, husbandry, and biosecurity variables significantly associated with the presence of *Campylobacter* spp. in small poultry flocks in Ontario, Canada (*n* = 138).

Variable	Category	OR	95% CI	*p*-Value
Species: turkey	No (*n* = 131)	Referent		
	Yes (*n* = 7)	16.89	1.08–263.49	0.044
Number of birds at risk	1–24 birds (*n* = 78)	Referent		
	25–49 birds (*n* = 31)	1.14	0.25–5.25	0.862
	50–260 birds (*n* = 29)	35.87	3.09–415.84	0.004
Pest control: any method ^1^	No (*n* = 63)	Referent		
	Yes (*n* = 75)	0.36	0.10–1.25	0.107
Number of birds at risk * Pest control ^2^	1-24 birds * no pest control (*n* = 41)	Referent		
	25–49 birds * pest control (*n* = 19)	2.55	0.30–21.94	0.393
	50–260 birds * pest control (*n* = 19)	0.04	0.002–0.73	0.03
Medication use within the last 12 months: antibiotics	No (*n* = 89)	Referent		
	Yes (*n* = 49)	0.19	0.06–0.59	0.004
Bird housing type	Inside only (*n* = 40)	Referent		
	Inside with some free-range access (*n* = 73)	0.28	0.10–0.80	0.017
	Free-range only (*n* = 25)	0.29	0.08–1.09	0.067
Mixed group with different species and/or types of birds	No (*n* = 92)	Referent		
	Yes (*n* = 46)	4.61	1.63–13.05	0.004

Overall *p*-value for the model: ≤0.001. Abbreviations: OR, odds ratio; CI, confidence interval. ^1^ Pest control: one or more pest control measures were in place for rodents, flies, beetles, and other pests. ^2^ Interaction between the number of birds at risk and pest control.

**Table 4:** Statistically significant (*p* ≤ 0.05) contrasts for the number of birds at risk and pest control interaction for the multivariable logistic regression farm model of demographic, husbandry, and biosecurity variables associated with the presence of *Campylobacter* spp. in small poultry flocks in Ontario, Canada (*n* = 138).

Contrast	OR	95% CI	*p*-Value
1–24 birds at risk * pest control (*n* = 37)	Referent		
50–260 birds at risk * no pest control (*n* = 10)	100.35	8.16–1234.49	≤0.001
25–49 birds at risk * no pest control (*n* = 12)	Referent		
50–260 birds at risk * no pest control (*n* = 10)	31.34	2.09–471.08	0.013
25–49 birds at risk * pest control (*n* = 19)	Referent		
50–260 birds at risk * no pest control (*n* = 10)	34.35	2.60–453.83	0.007
50–260 birds at risk * pest control (*n* = 19)	Referent		
50–260 birds at risk * no pest control (*n* = 10)	72.29	5.19–1006.43	0.001

Abbreviations: OR, odds ratio; CI, confidence interval.

**Table 5:** Coop model: Univariable analysis of indoor housing-related variables in a study investigating factors associated with the presence of *Campylobacter* spp. in small poultry flocks in Ontario, Canada. Variables with a *p*-value of ≤0.2 (shown in bold) were considered for further analyses.

Variable Description	Category	OR	95% CI	*p*-Value
Bedding type: soft-wood shavings (*n* = 124)	No (*n* = 37)	Referent		
	Yes (*n* = 87)	0.45	0.20–0.99	**0.048**
Bedding type: hard-wood shavings (*n* = 124)	No (*n* = 110)	Referent		
	Yes (*n* = 14)	1.01	0.32–3.23	0.985
Bedding type: sand (*n* = 124)	No (*n* = 109)	Referent		
	Yes (*n* = 15)	0.11	0.01–0.86	**0.036**
Bedding type: straw (*n* = 124)	No (*n* = 70)	Referent		
	Yes (*n* = 54)	1.5	0.71–3.15	0.284
Frequency of cleaning and/ or disinfecting barn/shed/coop ^1^ (*n* = 105)	Fairy frequently (*n* = 62)	Referent		
	Infrequently (*n* = 43)	0.94	0.41–2.15	0.888
Frequency of removing soiled litter and/or fecal material from barn/shed/coop (*n* = 102)	Daily (*n* = 23)	Referent		
	Weekly (*n* = 45)	1.67	0.57–4.86	0.346
	Monthly (*n* = 24)	1.14	0.33–3.90	0.831
	Yearly or never (*n* = 10)	0.57	0.09–3.41	0.539
Wear dedicated shoes when entering barn/shed/coop (*n* = 122)	No (*n* = 75)	Referent		
	Yes (*n* = 47)	0.49	0.22–1.08	**0.078**
Wear dedicated shoes when cleaning barn/shed/coop (*n* = 121)	No (*n* = 63)	Referent		
	Yes (*n* = 58)	0.63	0.30–1.34	0.233
Wear dedicated clothing when entering barn/shed/coop (*n* = 111)	No (*n* = 84)	Referent		
	Yes (*n* = 27)	0.23	0.07–0.73	**0.012**
Wear dedicated clothing when cleaning barn/shed/coop (*n* = 116)	No (*n* = 76)	Referent		
	Yes (*n* = 40)	0.48	0.21–1.13	**0.094**
Wear PPE ^2^ when entering barn/shed/coop (*n* = 118)	No (*n* = 70)	Referent		
	Yes (*n* = 48)	0.44	0.20–0.996	**0.049**
Wear PPE ^2^ when cleaning barn/shed/coop (*n* = 120)	No (*n* = 61)	Referent		
	Yes (*n* = 59)	0.58	0.27–1.25	**0.164**
Wear PPE ^2^ when entering and/or cleaning barn/shed/coop (*n* = 118)	No (*n* = 58)	Referent		
	Yes (*n* = 60)	0.52	0.24–1.12	**0.096**
Visitors allowed into barn/shed/coop (*n* = 123)	No (*n* = 49)	Referent		
	Yes (*n* = 74)	1.38	0.64–2.97	0.411
Visitors allowed into barn/shed/coop (*n* = 123)	No (*n* = 49)	Referent		
	Yes, required to wear dedicated clothing (*n* = 6)	1.13	0.19–6.88	0.892
	Yes, not required to wear dedicated clothing (*n* = 68)	1.4	0.64–3.06	0.395

Abbreviations: OR, odds ratio; CI, confidence interval; PPE, personal protective equipment. ^1^ Fairly frequently (when flock owners reported cleaning after each flock or cleaning more than once a year), infrequently (when flock owners reported cleaning once a year, less than once a year, or as needed). ^2^ PPE includes dedicated shoes and/or clothing.

## Data Availability

All relevant data are within the manuscript.
